# A new species of freshwater crab of the genus *Nanhaipotamon* Bott, 1968 (Crustacea, Decapoda, Brachyura, Potamidae) from Longhai, Fujian Province, China

**DOI:** 10.3897/zookeys.1062.71171

**Published:** 2021-10-12

**Authors:** Mao-Rong Cai, Qi-Hong Tan, Jie-Xin Zou

**Affiliations:** 1 Center for Disease Control and Prevention of Zhangzhou, Zhangzhou City, Fujian Province, China; 2 Research Laboratory of Freshwater Crustacean Decapoda & Paragonimus, School of Basic Medical Sciences, Nanchang University, Nanchang City, Jiangxi Province, China; 3 Key Laboratory of Poyang Lake Environment and Resource Utilization, Ministry of Education, Nanchang University, Nanchang City, Jiangxi Province, China

**Keywords:** freshwater crab, new species, Oriental region, taxonomy

## Abstract

A new species of freshwater crab of the genus *Nanhaipotamon* Bott, 1968 is described from Xiaye Village, Chengxiang Town, Longhai County, Zhangzhou City, Fujian Province, China. The new species is distinguished from congeners by the combination of characters of its carapace, third maxilliped, unequal chelipeds, triangular male abdomen and unique male first gonopod. Molecular evidence derived from partial mitochondrial 16S rRNA and COI genes also support the species as new.

## Introduction

The genus *Nanhaipotamon* Bott, 1968 was originally established by Bott (1968), with several species from Taiwan, Philippines, and the Ryukyus (Japan). Dai (1997) revised the genus and described nine species from China. The latest research on *Nanhaipotamon* described a new species from Macau, *N.macau* Huang, Wong & Ahyong, 2018 (Huang et al. 2018a). Currently, *Nanhaipotamon* is only known from Guangdong, Fujian, Zhejiang, Taiwan, Hong Kong, and Macau (Shih et al. 2011; Huang et al. 2018a). Prior to the present study, *Nanhaipotamon* contained 18 species (Dai 1999; Cheng et al. 2003; Shih et al. 2005; Cheng et al. 2009; Huang et al. 2012, 2018a; Lin et al. 2012; Lin et al. 2013).

In 2019, during a survey of freshwater crab resources in Longhai, Fujian Province, the first author collected several specimens of the genus *Nanhaipotamon*. In August 2020, we made another collection trip to obtain additional samples. After morphological comparison, we found the Longhai specimens to be distinct from known species of *Nanhaipotamon*. Molecular evidence based on the 16S rRNA and COI genes also support it as new. Therefore, we herein describe a new species, *Nanhaipotamonlonghaiense* sp. nov.

## Materials and methods

Specimens were collected from Longhai, Fujian Province by Mao-Rong Cai and preserved in 95% ethanol. The holotype and allotype were deposited at the Department of Parasitology of the Medical College of Nanchang University, Jiangxi, China (NCU MCP). Other examined materials were deposited at the Center for Disease Control and Prevention of Zhangzhou City, Zhangzhou, China (ZZCDC) and the National Tropical Disease Research Center, Shanghai, China (TDRC). Carapace width and length were measured in millimeters. The abbreviations G1 and G2 refer to the first and second gonopod. The terminology used herein primarily follows that of Dai (1999) and Davie et al. (2015).

We compared the new species with type materials of other nine species of *Nanhaipotamon* deposited in Chinese Academy of Sciences, Beijing, China (CAS CB). Comparative materials are as follows: *Nanhaipotamonguangdongense* Dai, 1997: holotype, 1♂, Guangdong Province, CAS CB 05141. *Nanhaipotamonhepingense* Dai, 1997: holotype, 1♂, Guangdong Province, Heping County, 7 May 1965, CAS CB 05106. *Nanhaipotamonhongkongense* Shen, 1940: holotype, 1♂, Hongkong, Jun. 1991, CAS CB 05107. *Nanhaipotamonnanriense* Dai, 1997: holotype, 1♂, Fujian Province, Putian County, Nanri Island, 15 Nov. 1975, CAS CB 05103. *Nanhaipotamonpinghense* Dai, 1997: holotype, 1♂, Guangdong Province, Heping County, 7 May 1965, CAS CB 05132. *Nanhaipotamonpingyuanense* Dai, 1997: holotype, 1♂, Pingyuan County, Guangdong Province, Sep. 1983, CAS CB 05131. *Nanhaipotamonwenzhouense* Dai, 1997: holotype, 1♂, Wenzhou City, Zhejiang Province, 1979, CAS CB 05143. *Nanhaipotamonyongchuense* Dai, 1997: holotype, 1♂, Fujian Province, Yongchun County, 29 Jun. 1977, CAS CB 05104. *Nanhaipotamonhuaanense* Dai, 1997: holotype, 1♂, Huaan County, Fujian Province, 15 Jun. 1984, CAS CB 05105.

Institutional abbreviations used in the paper are as follows: CAS CB, Chinese Academy of Sciences, Beijing, China; NCHUZOOL, Zoological Collections of the Department of Life Science, National Chung Hsing University, Taichung, Taiwan; NCU MCP, Department of Parasitology of the Medical College of Nanchang University, Jiangxi, China; SYSBM, Sun Yat-sen Museum of Biology, Sun Yat-Sen University, Guangzhou, China; ZRC, Zoological Reference Collection of Lee Kong Chian Natural History Museum (formerly Raffles Museum of Biodiversity Research), National University of Singapore, Singapore.

Approximately 50 mg of muscle tissue was excised from ambulatory legs and chelipeds. Total genomic DNA was extracted from the tissues using the DP1902 Tissue Kit (BioTeKe Inc., Beijing, China) following the manufacturer’s protocol. Then, the 16S rRNA gene was amplified using polymerase chain reaction (PCR) with the primers 1471 (5’-CCTGTTTANCAAAAACAT-3’) and 1472 (5’-AGATAGAAACCAACCTGG-3’) (Crandall and Fitzpatrick 1996). The COI gene was amplified with primers LCO1490 and HCO2198 (Folmer et al. 1994). The PCR conditions were as follows: denaturation for 50 s at 94 °C, annealing for 40 s at 52 °C and extension for 1 min at 72 °C (33 cycles), followed by a final extension for 10 min at 72 °C. The PCR products were purified and sequenced using an ABI 3730 automatic sequencer. We performed molecular analysis with the partial mitochondrial 16S rRNA and COI genes fragment. In total, 59 sequences were used to construct phylogenetic trees (Table 1). Sequences were aligned using MAFFT v. 7.215 (Katoh and Standley 2013) based on the G-INS-I method, and the conserved regions were selected with Gblocks 0.91b (Castresana 2000) using the default settings. The best-fitting model for Bayesian Inference (BI) analysis was determined by MrModeltest v. 2.3 (Nylander 2004), selected by the Akaike information criterion (AIC). The obtained model was GTR+G+I for both genes. MrBayes v. 3.2.6 (Ronquist et al. 2012) was employed to perform BI analysis, and four Monte Carlo Markov Chains of 2,000,000 generations were run with sampling every 1,000 generations. The first 500,000 generations were discarded as burn-in. The best evolutionary model for maximum likelihood (ML) analysis was HKY+I+G for 16S rRNA and GTR+G+I for COI, determined by MEGA X (Kumar et al. 2018) based on the Bayesian information criterion (BIC). An ML tree was built based on 1000 bootstrap replicates in MEGA X (Kumar et al. 2018). The pairwise distance based on the K2P (Kimura 2-Parameter) model was calculated by MEGA X (Kumar et al. 2018).

**Table 1.** GenBank accession number of the species used for phylogenetic analysis.

Species	Museum number	Locality	GenBank number	Reference
*Amamikuamamensis* Minei, 1973	NCHUZOOL 13125	Kagoshima, Japan	16S rRNA, AB428457	Shih et al. 2009
*Apotamonauteshainanensis* Parisi, 1916	NCHUZOOL	Hainan, China	16S rRNA, AB428459	Shih et al. 2009
*Candidiopotamonrathbunae* De Man, 1914	NCHUZOOL	Taiwan	16S rRNA, AB208598	Shih et al. 2006
*Cryptopotamonanacoluthon* Kemp, 1918	NCHUZOOL 13123	Taiwan	16S rRNA, AB428455	Shih et al. 2009
*Cantopotamonhengqinense* Huang, Ahyong & Shih, 2017	SYSBM 1559	Guangdong, China	16S rRNA, LC342047	Huang et al. 2017a
*Chinapotamonmaolanense* Zou, Bai & Zhou	NCU MCP 196101	Guizhou, China	16S rRNA, MH183299	Zou et al. 2018
*Chinapotamonglabrum* Dai, Song, Li & Liang, 1980	NCHUZOOL	Guangxi, China	16S rRNA, AB428451	Shih et al. 2009
*Diyutamoncereum* Huang, Shih & Ng, 2017	SYSBM 1555	Guizhou, China	16S rRNA, LC198519	Huang et al. 2017b
*D.cereum* Huang, Shih & Ng, 2017	SYSBM 1556	Guizhou, China	16S rRNA, LC198520	Huang et al. 2017b
*Geothelphusaalbogilva* Shy, Ng & Yu, 1994	NCHUZOOL	Taiwan	16S rRNA, AB127366	Shih et al. 2004
*G.marginatafulva* Naruse, Shokita & Shy, 2004	NCHUZOOL 13124	Okinawa, Japan	16S rRNA, AB428456	Shih et al. 2009
*G.olea* Shy, Ng & Yu, 1994	NCHUZOOL 13123	Taiwan	16S rRNA, AB428455	Shih et al. 2009
*Hainanpotamonfuchengense* Dai, 1995	NCHUZOOL 13128	Hainan, China	16S rRNA, AB428461	Shih et al. 2009
*Huananpotamonangulatum* Dai, Chen, Song, Fan, Lin & Zeng, 1979	NCHUZOOL	Fujian, China	16S rRNA, AB428454	Shih et al. 2009
*Luteomonspinapodum* Huang, Shih & Ahyong, 2018	SYSBM 001609	Guangdong, China	16S rRNA, LC383796	Huang et al. 2018a
*Minpotamonnasicum* Dai, Chen, Song, Fan, Lin & Zeng, 1979	NCHUZOOL 13121	Fujian, China	16S rRNA, AB428450	Shih et al. 2009
*Neotiwaripotamonjianfengense* Dai & Naiyanetr, 1994	NCHUZOOL 13127	Hainan, China	16S rRNA, AB428460	Shih et al. 2009
*Nanhaipotamonwupingense* Cheng, Yang, Zhang & Li, 2003	NCHUZOOL 13125	Fujian, China	16S rRNA, AB433548	Shih et al. 2011
*N.wupingense* Cheng, Yang, Zhang & Li, 2003	NCHUZOOL	Fujian, China	16S rRNA, AB470496	Shih et al. 2011
*N.pingyuanense* Dai, 1997	CAS CB 05131	Guangdong, China	16S rRNA, AB265237	Shih et al. 2007
*N.huaanense* Dai, 1997	CAS CB 05105	Fujian, China	16S rRNA, AB212870	Shih et al. 2005
*N.pinghense* Dai, 1997	CAS CB 05132	Guangdong, China	16S rRNA, AB433553	Shih et al. 2011
*N.hepingense* Dai, 1997	CAS CB 05106	Guangdong, China	16S rRNA, AB433552	Shih et al. 2011
*N.hongkongense* Shen, 1940	NCHUZOOL	Hongkong	16S rRNA, AB212869	Shih et al. 2005
*N.formosanum* Parisi, 1916	NCHUZOOL	Taiwan	16S rRNA, AB212867	Shih et al. 2005
*N.yongchuense* Dai, 1997	CAS CB 05104	Fujian, China	16S rRNA, AB433546	Shih et al. 2011
*N.nanriense* Dai, 1997	NCHUZOOL	Fujian, Chian	16S rRNA, AB212868	Shih et al. 2005
*N.wenzhouense* Dai, 1997	NCHUZOOL 13132	Zhejiang, China	16S rRNA, AB433543	Shih et al. 2011
*N.dongyinense* Shih, Chen & Wang, 2005	NCHUZOOL	Dongyin, Taiwan	16S rRNA, AB212863	Shih et al. 2005
*Qianguimonaflagellum* Dai, Song, Li & Liang, 1980	SYSBM 001404	Guangxi, China	16S rRNA, MG709239	Huang 2018.
*Ryukyumyaeyamense* Minei, 1973	NCHUZOOL 13126	Okinawa, Japan	16S rRNA, AB428458	Shih et al. 2009
*Socotrapotamonnojidense* Apel & Brandis, 2000	ZRC 2000.2232	Socota, Yemen	16S rRNA, AB428493	Shih et al. 2009
*Yarepotamongracilipa* Dai, Song, Li & Liang, 1980	ZRC	Guangxi, China	16S rRNA, AB428452	Shih et al. 2009
*N.longhaiense* sp. nov.	NCU MCP 417701	Fujian, China	16S rRNA, MT809486	This study
*N.longhaiense* sp. nov.	NCU MCP 417702	Fujian, China	16S rRNA, MT809487	This study
*N.longhaiense* sp. nov.	NCU MCP 417703	Fujian, China	16S rRNA, MT809488	This study
*N.longhaiense* sp. nov.	NCU MCP 417704	Fujian, China	16S rRNA, MT809489	This study
*Huananpotamonnanchengense* Dai, Zhou & Peng, 1995	NCHUZOOL	Jiangxi, China	COI, AB511392	Shih et al. 2011
*N.huaanense* Dai, 1997	CAS CB 05105	Fujian, China	COI, AB433572	Shih et al. 2011
*N.pingyuanense* Dai, 1997	CAS CB 05131	Guangdong, China	COI, AB265249	Shih et al. 2011
*N.wupingense* Cheng, Yang, Zhang & Li, 2003	NCHUZOOL 13125	Fujian, China	COI, AB433569	Shih et al. 2011
*N.hongkongense* Shen, 1940	NCHUZOOL	Hongkong	COI, AB433574	Shih et al. 2011
*N.yongchuense* Dai, 1997	CAS CB 05104	Fujian, China	COI, AB433567	Shih et al. 2011
*N.nanriense* Dai, 1997	NCHUZOOL	Fujian, Chian	COI, AB433565	Shih et al. 2011
*N.wenzhouense* Dai, 1997	NCHUZOOL 13132	Zhejiang, China	COI, AB433564	Shih et al. 2011
*N.dongyinense* Shih, Chen & Wang, 2005	NCHUZOOL	Dongyin, Taiwan	COI, AB433562	Shih et al. 2011
*N.formosanum* Parisi, 1916	NCHUZOOL	Taiwan	COI, AB433557	Shih et al. 2011
*N.guangdongense* Dai, 1997	–	Guangdong, China	COI, MK226145	Huang et al. 2018a
*N.macau* Huang, Wong & Ahyong, 2018	–	Macau	COI, MK226142	Huang et al. 2018a
*N.longhaiense* sp. nov.	NCU MCP 417701	Fujian, China	COI, MW703830	This study
*N.longhaiense* sp. nov.	NCU MCP 417702	Fujian, China	COI, MW729699	This study
*N.longhaiense* sp. nov.	NCU MCP 417703	Fujian, China	COI, MW729700	This study
*N.longhaiense* sp. nov.	NCU MCP 417704	Fujian, China	COI, MW729701	This study
*N.longhaiense* sp. nov.	NCU MCP 417705	Fujian, China	COI, MW729702	This study
*N.longhaiense* sp. nov.	NCU MCP 417706	Fujian, China	COI, MW729703	This study
*N.longhaiense* sp. nov.	NCU MCP 417707	Fujian, China	COI, MW729704	This study

## Results

### Systematics

**Family Potamidae Ortmann, 1896**

#### *Nanhaipotamon* Bott, 1968

##### Nanhaipotamon longhaiense sp. nov.

Taxon classificationAnimaliaDecapodaPotamidae

513E52FB-313A-54B6-A173-0860B44EB5BB

http://zoobank.org/E25133A7-AB4A-4CAA-8DF8-2DC9957384D9

**Figure 1.**
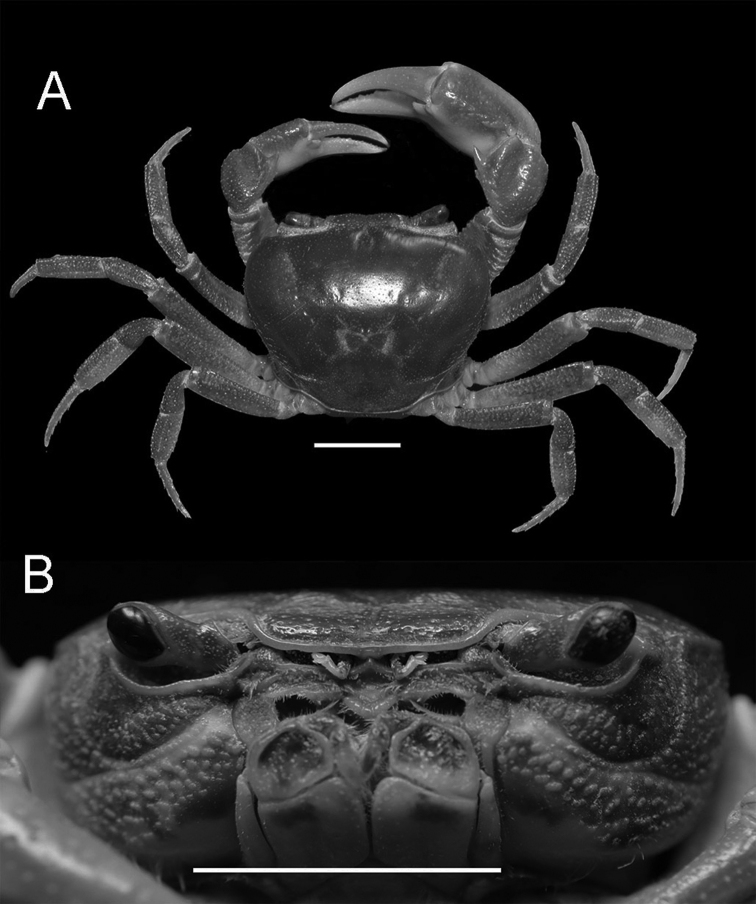
*Nanhaipotamonlonghaiense* sp. nov. Holotype male (25.2 × 21.5 mm) (NCU MCP 417701). **A** overall habitus **B** frontal view of cephalothorax. Scale bars: 1 cm.

**Figure 2.**
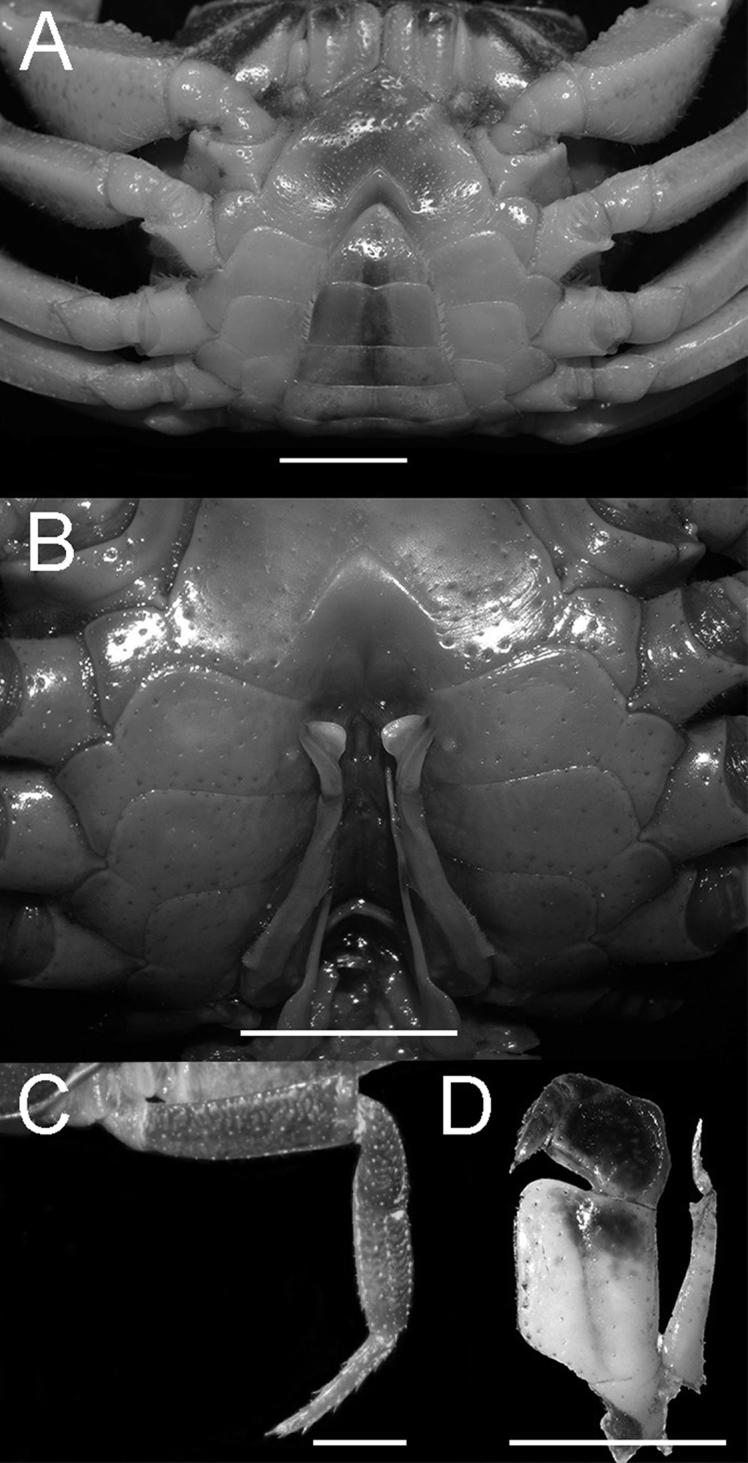
*Nanhaipotamonlonghaiense* sp. nov. holotype male (25.2 × 21.5 mm) (NCU MCP 417701) **A** ventral view of anterior thoracic sternum, telson, and male pleonal somites 4–6 **B** ventral view of sterno-pleonal cavity with G1 *in situ***C** the fourth ambulatory leg **D** left third maxilliped. Scale bars: 5 mm.

**Figure 3.**
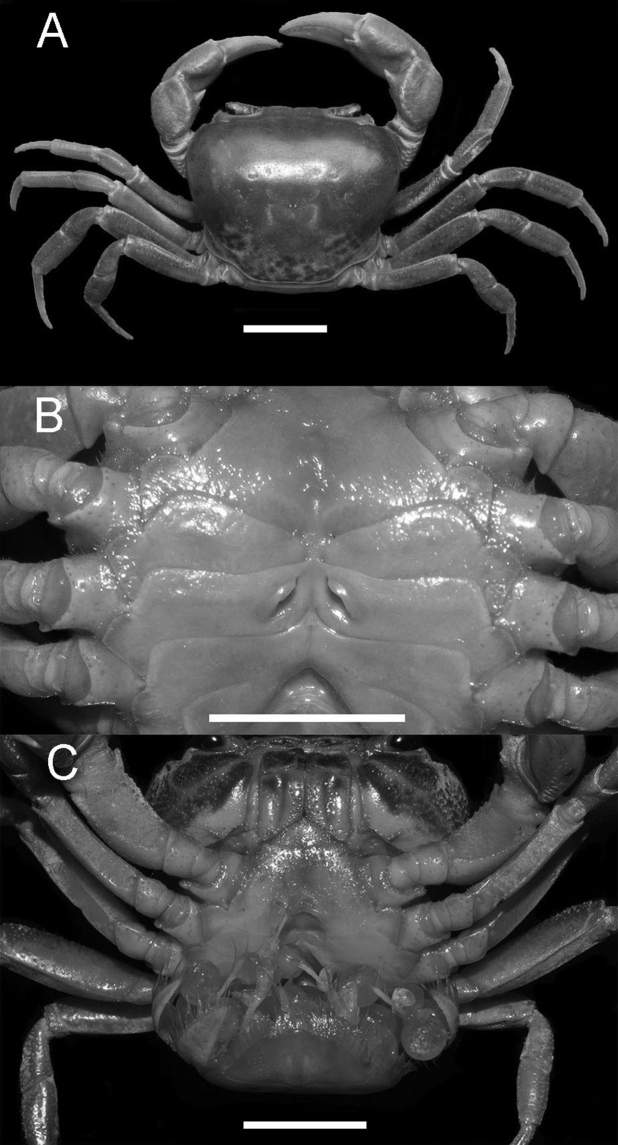
*Nanhaipotamonlonghaiense* sp. nov. Paratype female (26.5 × 22.5 mm) (NCU MCP 428601). **A** overall habitus **B** female vulvae **C** female holding eggs. Scale bars: 1 cm.

**Figure 4.**
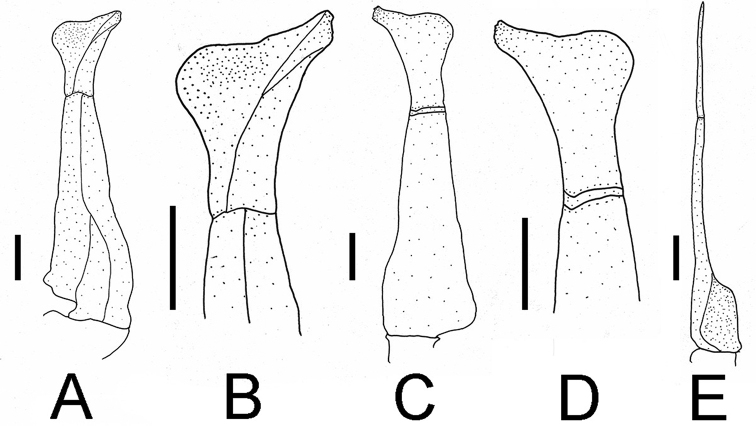
Gonopods of holotype **A** ventral view of left G1 **B** ventral view of terminal segment of left G1 **C** dorsal view of left G1 **D** dorsal view of terminal segment of left G1 **E** ventral view of left G2. Scale bars: 1 mm.

**Figure 5.**
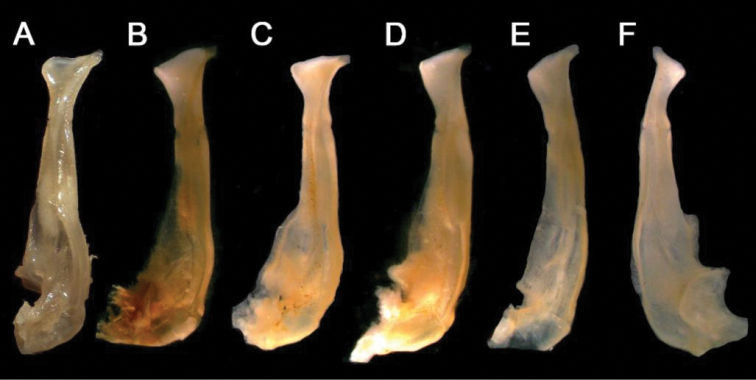
G1s of six species of *Nanhaipotamon*. **A***N.longhaiense* sp. nov., NCU MCP 417701 **B***N.guangdongense*, Dai, 1997, CB 05141 **C***N.hepingense*, Dai, 1997, CB 05106 **D***N.yongchuense*, Dai, 1997, CB 05104 **E***N.nanriense*, Dai, 1997, CB 05103 **F***N.huaanense*, Dai, 1997, CB 05105.

**Figure 6.**
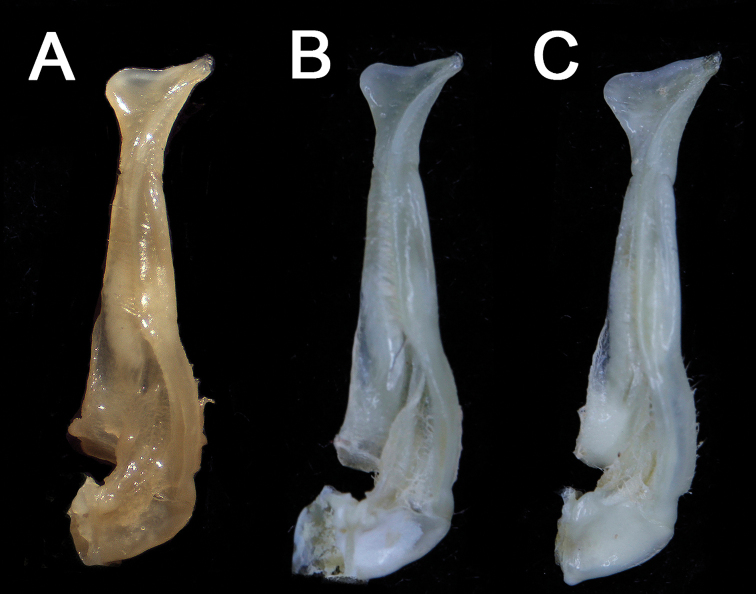
G1s of *N.longhaiense* sp. nov. **A** holotype, NCU MCP 417701 **B** paratype, ZZCDC 613201 **C** paratype, ZZCDC 613203.

**Figure 7.**
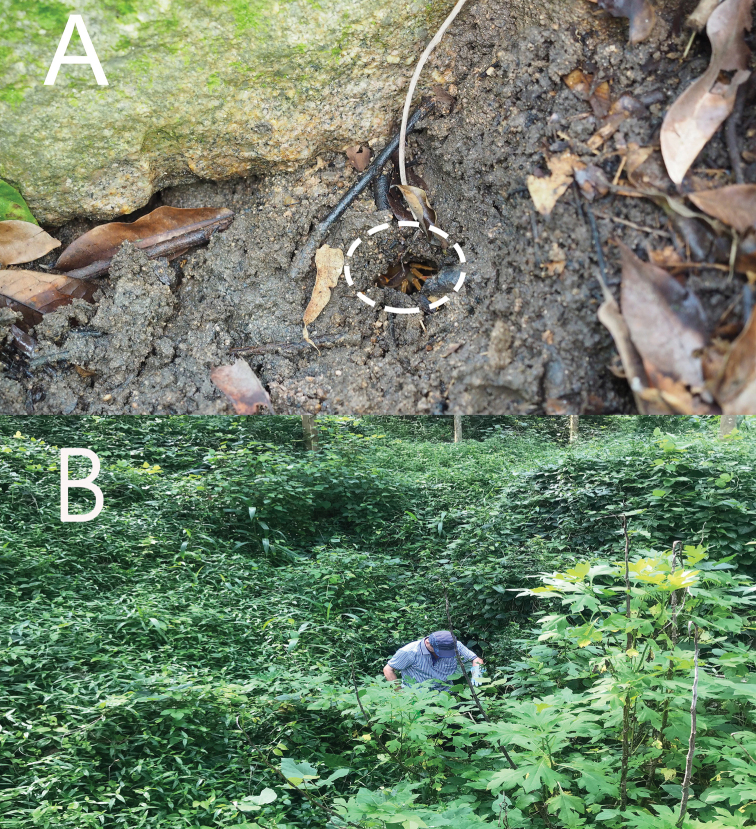
Habitat environment. **A** burrow inhabited by the new species (indicated by circle) **B** habitat environment.

Figs 1 , 2 , 3 , 4 , 5A , 6 , 7

###### Type material.

***Holotype***: ♂ (25.2 × 21.5 mm), China, Fujian Province, Longhai County, Chengxiang Town, Xiaye Village, 24°23'02"N, 117°34'76"E, alt. 55 m, 27 Aug. 2019, Mao-Rong Cai leg, NCU MCP 417701. ***Paratypes***: 1 ♀ (allotype) (26.5 × 22.5 mm), same data as holotype, NCU MCP 428601; 2 ♂♂ (27.1 × 22.0 mm, 29.0 × 23.3 mm), same data as for holotype, ZZCDC 613201, ZZCDC 613203.

###### Other specimens examined.

9 ♂♂ (28.1 × 22 .6 mm, 25.3 × 20.8 mm, 22.9 × 18.9 mm, 22.8 × 18.9 mm, 22.8 × 18.9 mm, 22.3 × 18.8 mm, 22.3 × 18.8 mm, 21.4 × 17.4 mm, 21.4 × 17.1 mm), same locality data as for holotype, 10 Aug. 2020, Mao-Rong Cai and Jie-Xin Zou leg, ZZCDC 613204 to 613208, TDRC 002101to 002104; 6 ♀♀ (26.4 × 22.2 mm, 23.4 × 18.9 mm, 21.6 × 17.7 mm, 21.2 × 16.8 mm, 21.2 × 16.2 mm, 18.4 × 15.2 mm), same locality data as for preceding, ZZCDC 613213 to 613215, TDRC 002105 to 002107.

###### Diagnosis.

Carapace subquadrate, regions indistinct, anterolateral regions slightly rugose; cervical groove shallow and wide, H-shaped groove shallow; postorbital cristae sharp, almost fused with epigastric cristae (Figs 1A, 3A). External orbital angle triangular, separated from anterolateral margin by wide, concave notch; epibranchial teeth small, granular; anterolateral margin lined with conspicuous granules (Figs 1A, 3A). Third maxilliped merus with shallow median depression, exopod flagellum slightly longer than 1/3 exopod length (Fig. 2D). Chelipeds strongly unequal; fingers with small gap when closed (Figs 1A, 3A). G1 slender, inner distal angle semicircular, inner margin of terminal segment convex, distal margin flat, outer distal angle blunt, laterally bent outwards at angle of about 60° (Figs 4A–D, 5A). Female vulvae ovate, medium-sized, wholly within sternite 6, opening directed inward (Fig. 3B).

###### Description.

Carapace subquadrate, broader than long; dorsal surface smooth, distinctly convex longitudinally, with tiny pits; anterolateral region rugose. Branchial regions swollen (Figs 1A, 3A). Cervical groove shallow and wide; H-shaped groove between gastric and cardiac regions shallow (Figs 1A, 3A). Epigastric cristae conspicuous, separated by narrow gap; postorbital cristae sharp, almost fused with epigastric cristae (Figs 1A, 3A). Front distinctly deflexed, margin ridged in dorsal view. External orbital angle triangular, separated from anterolateral margin by wide, concave notch. Epibranchial tooth small, granular. Anterolateral margin distinctly cristate, lined with approximately 20 granules (Figs 1A, 3A). Posterolateral surface smooth, with inconspicuous oblique striae, converging towards posterior carapace margin (Figs 1A, 3A). Orbits large; supraorbital, infraorbital margins cristate. Sub-orbital regions covered with granules (Fig. 1B); pterygostomial regions covered with large rounded granules; sub-hepatic regions covered with striae (Fig. 1B). Posterior margin of epistome with median triangle, lateral margin sinuous (Fig. 1B).

Third maxilliped merus about 1.2 times as broad as long, trapezoidal, with median depression; ischium about 1.3 times as long as broad, rectangular, with distinct median sulcus; exopod reaching approximately 1/4 of merus length, exopod flagellum slightly longer than 1/3 exopod length (Fig. 2D).

Chelipeds strongly unequal. Merus cross-section trigonal, inner-lower margin crenulated. Carpus surface weakly wrinkled, with longitudinal depression and sharp spine at inner-distal angle with spinule at base. Palm of larger chela about 1.3 times as long as high. Movable finger (dactylus) slightly shorter than the immovable finger (pollex). Inner margin of fingers with rounded, blunt teeth; fingers forming small gap when closed (Figs 1A, 3A).

Ambulatory legs slender, second leg longest, merus 0.5–0.6 times as long as carapace length; last leg with propodus 2.1 times as long as broad, slightly shorter than dactylus. Dactylus gently curved, with sharp spines on the surface (Figs 2C, 3A).

Male thoracic sternum smooth, pitted (Fig. 2A). Sternites 1, 2 completely fused to form triangular structure; sternites 2,3 separated by visible suture; sternites 3, 4 fused without obvious suture (Fig. 2A). Male sterno-pleonal cavity relatively deep, exceeding imaginary line connecting posterior edges of cheliped coxae (Fig. 2B). Median longitudinal suture of sternites 7, 8 deep and long. Tubercle of abdominal lock positioned at mid-length of sternite 5 (Fig. 2B). Female vulvae ovate, medium-sized, wholly within sternite 6, opening directed inward (Fig. 3B).

Male abdomen triangular; somites 4–6 gradually narrowed longitudinally, lateral margins slightly convex; somite 6 about 2.2 times as wide as long; telson about 1.4 times as wide as long (Fig. 2A).

G1 slender, tip of terminal segment reaches beyond pleonal locking tubercle (Fig. 2B), subterminal segment about 2.4 times as long as terminal segment (Fig. 4A). Inner distal angle semicircular, inner margin of terminal segment convex, distal margin flat, outer distal angle blunt, bent outwards at angle of about 60° (Figs 4A–D, 5A). G2 subterminal segment about 1.9 times length of distal segment (Fig. 4E).

###### Etymology.

The new species is named after the county where is located, Longhai County, Zhangzhou City, Fujian Province, China.

###### Distribution.

Longhai County, Zhangzhou City, Fujian Province, China.

###### Ecology.

The new species occurs in the wetlands of low-elevation hills and mountains, amongst dense vegetation where there is little to no water flow year-round (Fig. 7B). During the day, the crabs usually hide in mud burrows close to the water source (Fig. 7A) or hide under rocks under water. We observed a berried female in August, suggesting the time around this month to be a part of the breeding season (Fig. 3C).

###### Remarks.

With a convex carapace dorsal surface, unequal chelipeds and triangular male abdomen, *Nanhaipotamonlonghaiense* sp. nov. fits the diagnosis of *Nanhaipotamon*. Like some species within this genus, *N.longhaiense* sp. nov. shows intraspecific variation in G1 morphology, the distal margin of the G1 terminal segment is flat to oblique (Fig. 6A–C). In the holotype, the distal margin is flat (Fig. 6A), whereas in some adult specimens, the distal margin is oblique (Fig. 6B, C), and the inner margin of the G1 terminal segment is slightly convex to distinctly convex (Fig. 6A–C).

We make comparisons between the new species and seven species of *Nanhaipotamon*, among which *N.wuping* and *N.macau* are morphologically similar to this new species, *N.yongchuense*, *N.huaanense* and *N.nanriense* are geographically close (Dai 1999), and *N.guangdongense* and *N.hepingense* are from Guangdong near Fujian (Dai 1999). *Nanhaipotamonlonghaiense* sp. nov. can be differentiated from its congeners by its unique G1 (Fig. 5A). Compared to *N.longhaiense* sp. nov., which has a semicircular G1 terminal segment inner distal angle, *N.guangdongense*, *N.hepingense*, *N.yongchuense*, *N.nanriense*, and *N.huaanense* differ in having instead a bluntly triangular G1 terminal segment inner distal angle (Fig. 5B–F). The G1 terminal segment inner distal angle is also semicircular in both *N.wupingense* and *N.macau* (Huang et al. 2018a); however, the terminal segments in these two species are proportionately larger. In *N.macau*, the G1 terminal segment distal margin is sinuous to V-shaped (cf. Huang et al. 2018a: fig. 5D, E). In *N.wupingense*, the G1 terminal segment distal margin is sinuous to an inverte V-shaped (cf. Cheng et al. 2003: fig. 7; Huang et al. 2018a: fig. 6D). In *N.longhaiense* sp. nov., however, the G1 terminal segment distal margin is flat to oblique (Fig. 6A–C). The detailed differences between the new species and congeners are presented in Table 2.

**Table 2.** Morphological difference among eight species of *Nanhaipotamon*.

Species/character	Ratio of flagellum length to exopod length	G1 *in situ*	Inner margin of G1 terminal segment	Inner distal angle of G1 terminal segment	Outer distal angle of G1 terminal segment
*longhaiense* sp. nov.	0.4 (Fig. 2D)	Exceeding pleonal locking tubercle (Fig. 2B)	Convex (Fig. 5A)	Semicircular (Fig. 5A)	Relatively stout; bent outwards at angle of about 60° (Fig. 5A)
*N.nanriense* (cf. Dai 1999: fig. 53)	0.4	Exceeding pleonal locking tubercle	Gently convex (Fig. 5E)	Blunt; triangular (Fig. 5E)	Relatively stout; bent outwards at angle of about 45° (Fig. 5E)
*N.yongchuense* (cf. Dai 1999: fig. 54)	0.1	Exceeding pleonal locking tubercle	Gently convex (Fig. 5D)	Blunt; triangular (Fig. 5D)	Relatively stout; bent outwards at angle of about 45° (Fig. 5D)
*N.huaanense* (cf. Dai 1999: fig. 55)	0.1	Reaching pleonal locking tubercle	Gently convex	Blunt; triangular	Relatively slender; bent outwards at angle of about 60°
*N.wupingense*	0.1 (cf. Cheng et al. 2003: fig. 5)	Exceeding pleonal locking tubercle (cf. Cheng et al. 2003: fig. 3)	Gently convex (cf. Cheng et al. 2003: fig. 7)	Distinctly expanded; semicircular (cf. Cheng et al. 2003: fig. 7)	Relatively stout; bent outwards >60° (cf. Cheng et al. 2003: fig. 7)
*N.macau*	0.2 (cf. Huang et al. 2018a: fig. 5A)	Exceeding pleonal locking tubercle (cf. Huang et al. 2018a: fig. 3D)	Gently convex (cf. Huang et al. 2018a: fig. 5D, E)	Distinctly expanded; semicircular (cf. Huang et al. 2018a: fig. 5D, E)	Relatively stout; bent outwards at angle of about 90° (cf. Huang et al. 2018a: fig. 5D, E)
*N.hepingense* (cf. Dai 1999: fig. 59)	0.5	Exceeding suture 4/5	Gently convex (Fig. 5C)	Blunt; triangular (Fig. 5C)	Relatively stout; bent outwards >60° (Fig. 5C)
*N.guangdongense* (cf. Dai 1999: fig. 60)	0.5	Not reaching pleonal locking tubercle	Distinctly convex (Fig. 5B)	Triangular (Fig. 5B)	Relatively stout; bent outwards >60° (Fig. 5B)

### Phylogenetic analyses

**Figure 8.**
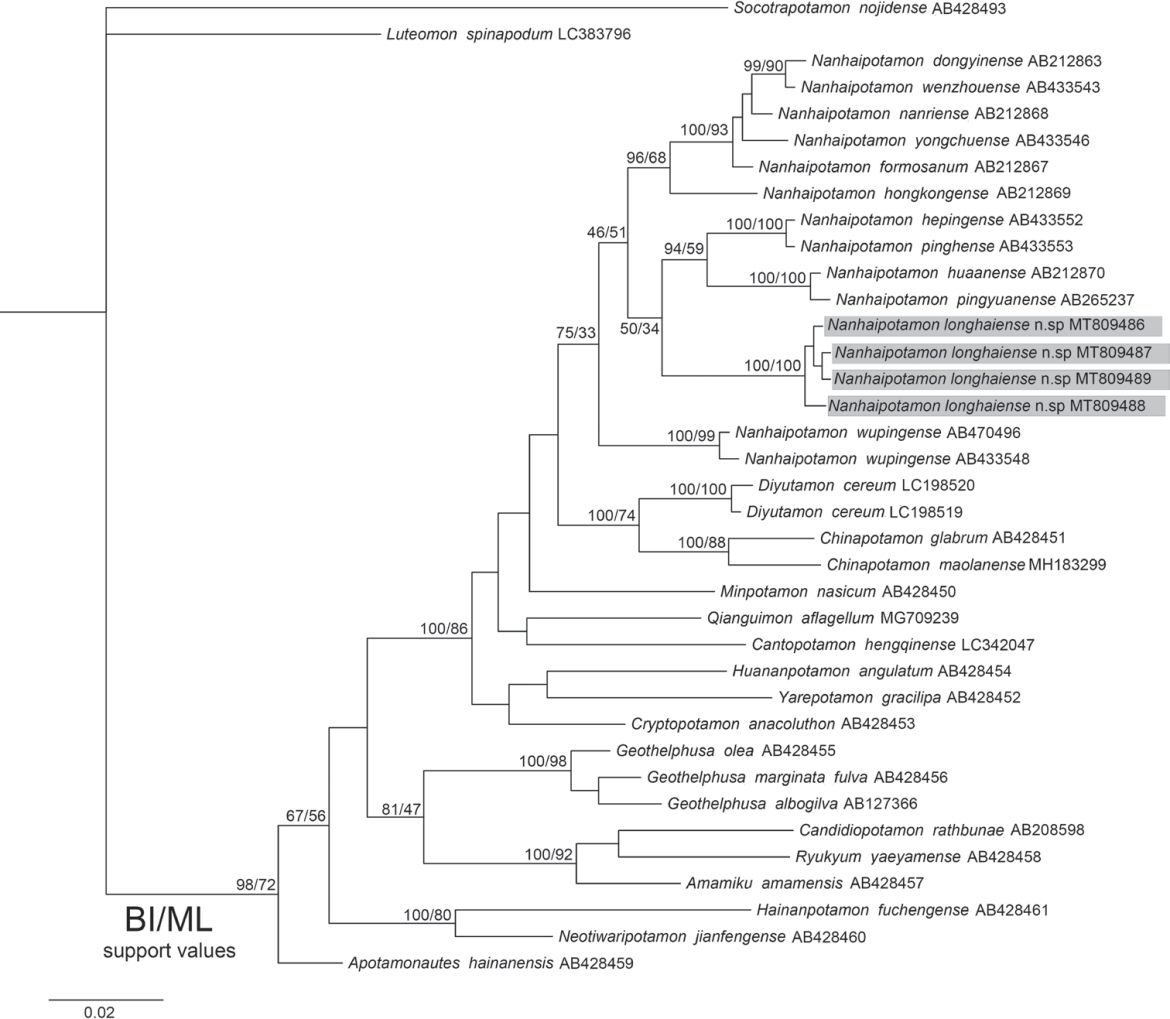
Phylogenetic tree based on 16S rRNA. Topologies and branch lengths were obtained from BI analysis. Support values represented at the nodes were from BI and ML.

**Figure 9.**
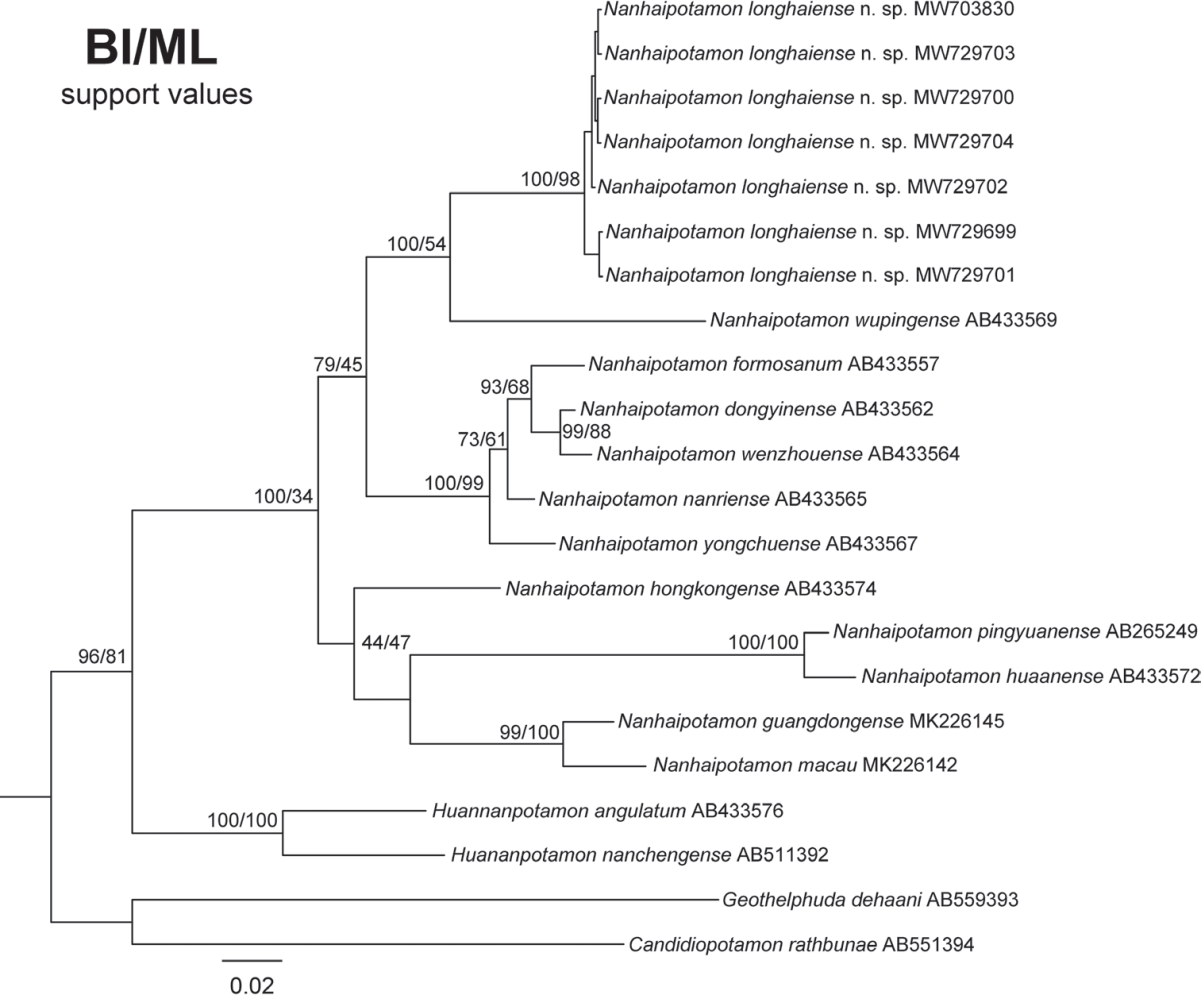
Phylogenetic tree based on COI. Topologies and branch lengths were obtained from BI analysis. Support values represented at the nodes were from BI and ML.

In this study, we obtained the partial mitochondrial 16S rRNA and COI genes from specimens of *Nanhaipotamon* collected from Xiaye Village, Chengxiang Town, Longhai County, Fujian Province, China. A total of 37 546 bp 16S rRNA gene sequences and 22 658 bp COI gene sequences were used to construct the BI and ML trees. The topological structures of the 16S rRNA and COI trees are similar. Both trees show that *N.longhaiense* sp. nov. and 11 other species of *Nanhaipotamon* are clustered into one clade (Figs 8, 9). In the 16S rRNA tree, four sequences of *N.longhaiense* sp. nov. form a small branch within *Nanhaipotamon*, while in the COI tree, the *N.longhaiense* sp. nov. clade and *N.wupingense* are sister to each other, indicating a close phylogenetic relationship between *N.longhaiense* sp. nov. and *N.wupingense*. The pairwise distances between the 12 species of *Nanhaipotamon* were calculated based on the COI gene. The result shows that the pairwise genetic distances between *Nanhaipotamon* range from 0.0239 to 0.1552 (Table 3), while distances between *N.longhaiense* sp. nov. and its congeners are from 0.0880 to 0.1423. Therefore, the genetic distance is large enough to support *N.longhaiense* sp. nov. as new. Both the phylogenetic position and genetic divergences provide evidence supporting the recognition of *N.longhaiense* sp. nov. as a new species.

**Table 3.** Pairwise genetic distances between 12 species of *Nanhaipotamon*.

Species	1	2	3	4	5	6	7	8	9	10	11	12
*N.formosanum*												
*N.dongyinense*	0.0269											
*N.wenzhouense*	0.0319	0.0124										
*N.nanriense*	0.0303	0.0255	0.0306									
*N.yongchuense*	0.0458	0.0408	0.0425	0.0305								
*N.hongkongense*	0.1088	0.1009	0.1031	0.0928	0.0991							
*N.pingyuanense*	0.1552	0.1272	0.1340	0.1437	0.1390	0.1444						
*N.huaanense*	0.1503	0.1227	0.1317	0.1437	0.1366	0.1444	0.0239					
*N.guangdongense*	0.1243	0.1140	0.1207	0.1098	0.1302	0.0985	0.1373	0.1420				
*N.macau*	0.1306	0.1246	0.1275	0.1159	0.1342	0.1066	0.1437	0.1461	0.0409			
*N.wupingense*	0.1116	0.0975	0.1058	0.1039	0.1141	0.1018	0.1529	0.1529	0.1366	0.1534		
*N.longhaiense* sp. nov.	0.0976	0.0880	0.0920	0.0922	0.0902	0.1031	0.1423	0.1329	0.1184	0.1252	0.1033	

## Discussion

*Nanhaipotamon* is endemic to China and mainly distributed in the low-elevation coastal areas or islands in southeastern China. Due to the isolating effect of mountain ranges, *Nanhaipotamon* is restricted to an area east of the Wuyishan Range and south of the Nanling Range (Shih et al. 2011). With 18 species, including *N.longhaiense* sp. nov., species diversity in *Nanhaipotamon* is the highest among sympatric genera (*Longpotamon*, *Somanniathelphusa*, *Huananpotamon*, *Bottapotamon*, *Minpotamon*, *Heterochelamon*, *Cantopotamon*, *Cryptopotamon*, *Eurusamon*, *Yarepotamon*, *Yuebeipotamon*) except *Geothelphusa*. *Huananpotamon* is followed by *Nanhaipotamon*, with 15 species distributed on both sides of the Wuyishan Range (Fujian and Jiangxi Provinces) (Shih et al. 2011; Chu et al. 2018). While all the other sympatric genera consist of fewer than 10 species. Therefore, *Nanhaipotamon* has important value as part of the regional biodiversity.

In the morphological classification of freshwater crabs, the G1 character provide important morphological identification features (Dai 1999). Intraspecific variation in G1 morphology has been reported in some species of *Nanhaipotamon*, such as *N.guangdongense* from different localities (Huang et al. 2012; Huang et al. 2018a). In *N.longhaiense* sp. nov., intraspecific variation of G1 morphology was also found. Several questions have arisen due to G1 intraspecific variation: Dai (1997) described *N.hepingense* and *N.pinghense*, both from Heping County, Guangdong Province. Shih et al. (2011) provided molecular evidence that they are synonymous and many scholars agree with this (Huang et al. 2012; Chu et al. 2018). Huang et al. (2012) described *N.zhuhaiense* in Zhuhai, Guangdong Province, where *N.guangdongense* is also found. Later, Huang et al. (2018a) indicated that *N.zhuhaiense* and *N.guangdongense* are probably conspecific, but they did not have sufficient material on which to take taxonomic action. These problems were caused by intraspecific variation, which makes it difficult to classify species based on morphology alone. Therefore, when describing a new species of this genus, it is recommended that morphological classification be combined with molecular analysis. There are likely other problems with some species in this genus, and therefore a revision is necessary.

## Conclusion

In this article, we report a new species of *Nanhaipotamon* collected from Xiaye Village, Chengxiang Town, Longhai County, Fujian Province, China. *Nanhaipotamonlonghaiense* sp. nov. can be distinguished from congeners by the combination of carapace, third maxilliped, and male first gonopod characters. Molecular evidence based on the mitochondrial 16S rRNA and COI genes also support it as a new species of the genus *Nanhaipotamon*.

## Supplementary Material

XML Treatment for Nanhaipotamon longhaiense

## Supplementary materials

Supplementary material 1 BI phylogenetic tree based on 16S gene Data type phylogenetic https://binary.pensoft.net/file/598460This dataset is made available under the Open Database License (http://opendatacommons.org/licenses/odbl/1.0/). The Open Database License (ODbL) is a license agreement intended to allow users to freely share, modify, and use this Dataset while maintaining this same freedom for others, provided that the original source and author(s) are credited.Mao-Rong Cai, Qi-Hong Tan, Jie-Xin Zou

Supplementary material 2 ML phylogenetic tree based on 16S gene Data type phylogenetic https://binary.pensoft.net/file/598461This dataset is made available under the Open Database License (http://opendatacommons.org/licenses/odbl/1.0/). The Open Database License (ODbL) is a license agreement intended to allow users to freely share, modify, and use this Dataset while maintaining this same freedom for others, provided that the original source and author(s) are credited.Mao-Rong Cai, Qi-Hong Tan, Jie-Xin Zou

Supplementary material 3 BI phylogenetic tree based on COI gene Data type phylogenetic https://binary.pensoft.net/file/598462This dataset is made available under the Open Database License (http://opendatacommons.org/licenses/odbl/1.0/). The Open Database License (ODbL) is a license agreement intended to allow users to freely share, modify, and use this Dataset while maintaining this same freedom for others, provided that the original source and author(s) are credited.Mao-Rong Cai, Qi-Hong Tan, Jie-Xin Zou

Supplementary material 4 ML phylogenetic tree based on COI gene Data type phylogenetic https://binary.pensoft.net/file/598463This dataset is made available under the Open Database License (http://opendatacommons.org/licenses/odbl/1.0/). The Open Database License (ODbL) is a license agreement intended to allow users to freely share, modify, and use this Dataset while maintaining this same freedom for others, provided that the original source and author(s) are credited.Mao-Rong Cai, Qi-Hong Tan, Jie-Xin Zou
